# A comparative analysis of face and object perception in 2D laboratory and virtual reality settings: insights from induced oscillatory responses

**DOI:** 10.1007/s00221-024-06935-3

**Published:** 2024-10-12

**Authors:** Merle Sagehorn, Joanna Kisker, Marike Johnsdorf, Thomas Gruber, Benjamin Schöne

**Affiliations:** 1 Experimental Psychology I, Institute of Psychology, Osnabrück University, Lise-Meitner-Str. 3, 49076 Osnabrück, Germany; 2 Department of Psychology, Norwegian University of Science and Technology, Trondheim, Norway

**Keywords:** Face perception, VR, Induced oscillations, Realistic conditions, EEG

## Abstract

In psychophysiological research, the use of Virtual Reality (VR) for stimulus presentation allows for the investigation of how perceptual processing adapts to varying degrees of realism. Previous time-domain studies have shown that perceptual processing involves modality-specific neural mechanisms, as evidenced by distinct stimulus-locked components. Analyzing induced oscillations across different frequency bands can provide further insights into neural processes that are not strictly phase-locked to stimulus onset. This study uses a simple perceptual paradigm presenting images of faces and cars on both a standard 2D monitor and in an immersive VR environment. To investigate potential modality-dependent differences in attention, cognitive load, and task-related post-movement processing, the induced alpha, theta and beta band responses are compared between the two modalities. No evidence was found for differences in stimulus-dependent attention or task-related post-movement processing between the 2D conditions and the realistic virtual conditions in electrode space, as posterior alpha suppression and re-synchronization of centro-parietal beta did not differ between conditions. However, source analysis revealed differences in the attention networks engaged during 2D and 3D perception. Midfrontal theta was significantly stronger in laboratory conditions, indicating higher cognitive load than in the VR environment. Exploratory analysis of posterior theta showed stronger responses in VR, possibly reflecting the processing of depth information provided only by the 3D material. In addition, the theta response seems to be generated by distinct neuronal sources under realistic virtual conditions indicating enhanced involvement of semantic information processing and social cognition.

**Supplementary Information:**

The online version contains supplementary material available at 10.1007/s00221-024-06935-3.

## Introduction

The ability to perceive and accurately interpret faces and objects is a fundamental feature of human cognition. Investigating visual information processing and the underlying neural processes thus serves as a gateway to understanding our perception of the world and the stimuli it contains. An important aspect of this world is its three-dimensionality and contextual congruence, to which the human brain is highly adapted. In research that aims to transfer these real-life characteristics to the laboratory Virtual Reality (VR) is often used to achieve realistic perceptual features, i.e., relative size, depth information, and spatial proximity, while maintaining the experimental control required for electrophysiological measures like electroencephalography (EEG) (e.g., Blascovich et al. 2002; Gabana et al. 2017; Gromer et al. 2018; Kisker et al. 2021a, b; Newman et al. 2022; Rubo and Gamer 2018; Schöne et al. 2023; Schöne et al. 2021; Van Den Oever et al. 2022). Despite the technical challenges posed by the use of head-mounted displays in conjunction with the sensitive EEG sensors (e.g., Cattan et al. 2018; Hertweck et al. 2019; Kisker et al. 2024; Tauscher et al. 2019; Weber et al. 2021), VR has been successfully implemented in EEG research to investigate the neural mechanisms underlying perceptual, attentional as well as mnemonic processes in realistic virtual settings (e.g., Johnsdorf et al. 2023a; Kisker et al. 2021a, b; Sagehorn et al. 2023, 2024; Schöne et al. 2023; Schöne et al. 2021).

Within the field of face perception research, the analysis of event-related potentials (ERPs) has provided valuable insights into the neural basis of face and object perception at different degrees of realism (e.g., Kirasirova et al. 2021; Sagehorn et al. 2023, 2024; Stolz et al. 2019). The analysis of early stimulus-specific components (Berchicci et al. 2016; Nasr 2010) indicates that the common face-related effects are limited to laboratory conditions (Sagehorn et al. 2023, 2024). In contrast, later and response-locked components show distinct amplitude differences between virtual 3D faces and objects that are absent for the 2D equivalents, suggesting modality-specific stimulus processing at a more conceptual level. This is further supported by the neural generators underlying these later components that reflect emotional and self-relevant processing only under realistic virtual conditions (Sagehorn et al. 2023). It remains an open question how the underlying neural mechanisms driving these effects adapt to the change in modality and whether they originate from the same neural networks.

To address this question, induced oscillatory responses provide a valuable tool as they offer insights into the way our brain processes and integrates information gathered from the external world (for review see e.g., Başar et al. 2000). In contrast to ERPs, induced oscillatory activity is not precisely coupled in time to the onset of the stimulus. Instead, the induced response is characterized by a variable latency across trials (Eckhorn et al. 1990) and is usually analyzed by averaging individual trials in the frequency domain, revealing information that would be obscured by averaging in the time domain (Gruber and Müller 2002; Kisker et al. 2024). Neuronal oscillations are therefore particularly suitable to investigate the neural mechanisms involved in visual perception and subsequent stimulus identification that are not strictly phase-locked to stimulus onset, including attention, cognitive load, and decision making and execution. Considering the differences between 2D monitor and 3D VR stimuli in terms of their basic perceptual properties but also their integration into a congruent context and their inherent meaning for the observer, an adaptation of these processes to the respective modality is to be expected. Therefore, an investigation of how the neural correlates associated with these processes are altered by a modality transfer is of high interest and relevance in the field of visual perception.

However, corresponding analyses in the frequency domain based on the direct comparison of neuronal oscillations between different modalities are still limited. With regard to cognitive processing, some studies investigated signal changes in the theta band as an index of cognitive load when transferring the respective experimental paradigms to 3D conditions with inconclusive results (Dan and Reiner 2017; Slobounov et al. 2015; Xu and Sui 2021). Where a paper folding task under virtual 3D conditions was found to reduce cognitive load compared to the 2D condition, as evidenced by a decrease in the theta band (Dan and Reiner 2017), the application of a more interactive spatial navigation task in a 3D VR environment resulted in higher frontal midline theta power as the 2D VR setting (Slobounov et al. 2015). Similarly, viewing 3D VR videos compared to 2D monitor videos of natural scenes also enhanced frontal theta responses while improving functional connectivity in the underlying neural networks (Xu and Sui 2021). In face perception research in particular, increased attention and emotion due to higher stimulus salience in VR environments has been found to manifest in greater desynchronization of the alpha band and beta band (Schubring et al. 2020).

The present study aims to investigate how the experimental modality, i.e., 2D laboratory versus 3D VR conditions, affects cognitive load, stimulus-dependent attention, and task-related movement processing using a simple face-car-discrimination paradigm (see also Sagehorn et al. 2024). Cars are often used as an object control category (e.g., Boehm et al. 2011; Dering et al. 2009; Kloth et al. 2013; Kuefner et al. 2010; Ratcliff et al. 2009; Thierry et al. 2007) due to their high everyday relevance and familiarity to the observer (Rossion and Jacques 2011). In addition, blurred images of both stimulus categories serve as standardly used perceptual controls (e.g., Bombari et al. 2013; Kuefner et al. 2010; Rossion 2014; Schwaninger et al. 2009), which retain the basic physical properties but reduce the semantic meaning of the stimulus category. By transferring both stimulus categories to 3D VR conditions, they are presented in their real-life proportions, wherein faces and cars differ significantly in both their physical characteristics and contextual relevance, much more so than in 2D and at the same size on a screen. Therefore, the stimulus-specific cognitive processing mechanisms may not transfer in the same way to the more realistic conditions.

Specifically, the attentional demands required for perception and identification of each stimulus category are investigated by means of the posterior induced alpha band response (iABR). Alpha band activity functions as the mechanism for suppressing the processing of irrelevant information, which in turn provides alpha suppression as an inverse index of increased attention (Başar et al. 2000; Klimesch et al. 1998; Köster and Gruber 2022). Potential differences in cognitive load associated with the discrimination task are explored based on the midfrontal induced theta band response (iTBR) as an objective index (Dan and Reiner 2017), as theta band responses have been reliably associated with cognitive capacity (e.g., Başar et al. 2000; Castro-Meneses et al. 2020; Klimesch 1999; Köster and Gruber 2022; Puszta 2022). To further illuminate the functional role of the iABR and the iTBR, the results from electrode space will be complemented by source reconstructions of relevant effects. The integrative process of decision making and execution associated with active stimulus identification can be addressed by examining the re-synchronization of induced beta band response (iBBR) after decision-related movement (e.g.; Cardellicchio et al. 2020; Cassim et al. 2001; Fischer et al. 2018; Neuper and Pfurtscheller 1996; Pfurtscheller et al. 1996).

As the presentation of persons and objects under realistic virtual conditions is closer to real-life scenarios than 2D laboratory conditions in terms of perceptual characteristics and congruent context embedding, we expected a lower cognitive load for the VR modality, which would be reflected in a lower midfrontal iTBR. This would be partly consistent with previous study results (Dan and Reiner 2017). Regarding the influences on attention, higher demands are plausible under VR conditions due to increased stimulus complexity and salience, which would be reflected in a stronger negative posterior iABR (Schubring et al. 2020). However, it is also possible to find higher demands in 2D due to the artificiality of the laboratory environment, which would be reflected in a stronger negative posterior iABR under 2D conditions. The investigation of differences in task-related post-movement processing between VR and 2D conditions remains exploratory but in conjunction with increased attention and stimulus saliency, it could be expected to observe a more avid task performance in immersive VR environments, reflected in an increased post-movement iBBR measured over centro-parietal cortical areas.

## Methods

This paper complements a previously published article (Sagehorn et al. 2024) reporting ERP results from the data of the same experimental setup and available under the Creative Commons Attribution License (CC BY). Both papers build on a previously conducted experiment on face perception under realistic virtual conditions using a similar experimental set up only using faces and two different perceptual control stimuli (i.e., blurred and scrambled faces; see Sagehorn et al. 2023).

### Participants

An a priori power analysis, performed using Power Contour Estimation as described by (Baker et al. 2020), was based on key parameters from our prior study (Sagehorn et al. 2023), i.e., mean differences, within- and between-subject standard deviations, and the trial count. This analysis determined that a total of 55 participants were required to detect the expected small to medium effects. To account for potential issues related to EEG acquisition and data quality, we initially recruited 65 participants from Osnabrück University’s student population. All participants underwent pre-experimental screening to assess the absence of psychological or neurological disorders and regular drug use. In cases where vision correction was necessary, participants were required to use contact lenses, as glasses may prove uncomfortable when worn under the EEG cap and VR headset for extended periods. Before recruitment, we ensured that participants had not been involved in the stimulus creation process and had not previously taken part in the earlier VR-based face perception study using some of the same stimuli. After participation, we also assured that the participants did not recognize any of the individuals they were shown. Informed written consent was obtained from all participants, and they received either partial course credits or a compensation of 18€ for their participation. The study was conducted in compliance with the Declaration of Helsinki and approved by the local ethics committee.

Subsequently, ten participants were excluded from the study, with reasons including unmet anamnesis criteria (*n* = 1), insufficient data quality (*n* = 7), or self-aborted experiments (*n* = 2). Ultimately, data from 55 participants were utilized for subsequent analyses, with an average age of 22.85 years (SD = 4.3 years), consisting of 38 females and 54 right-handed individuals.

### Stimulus material

**Fig. 1:**
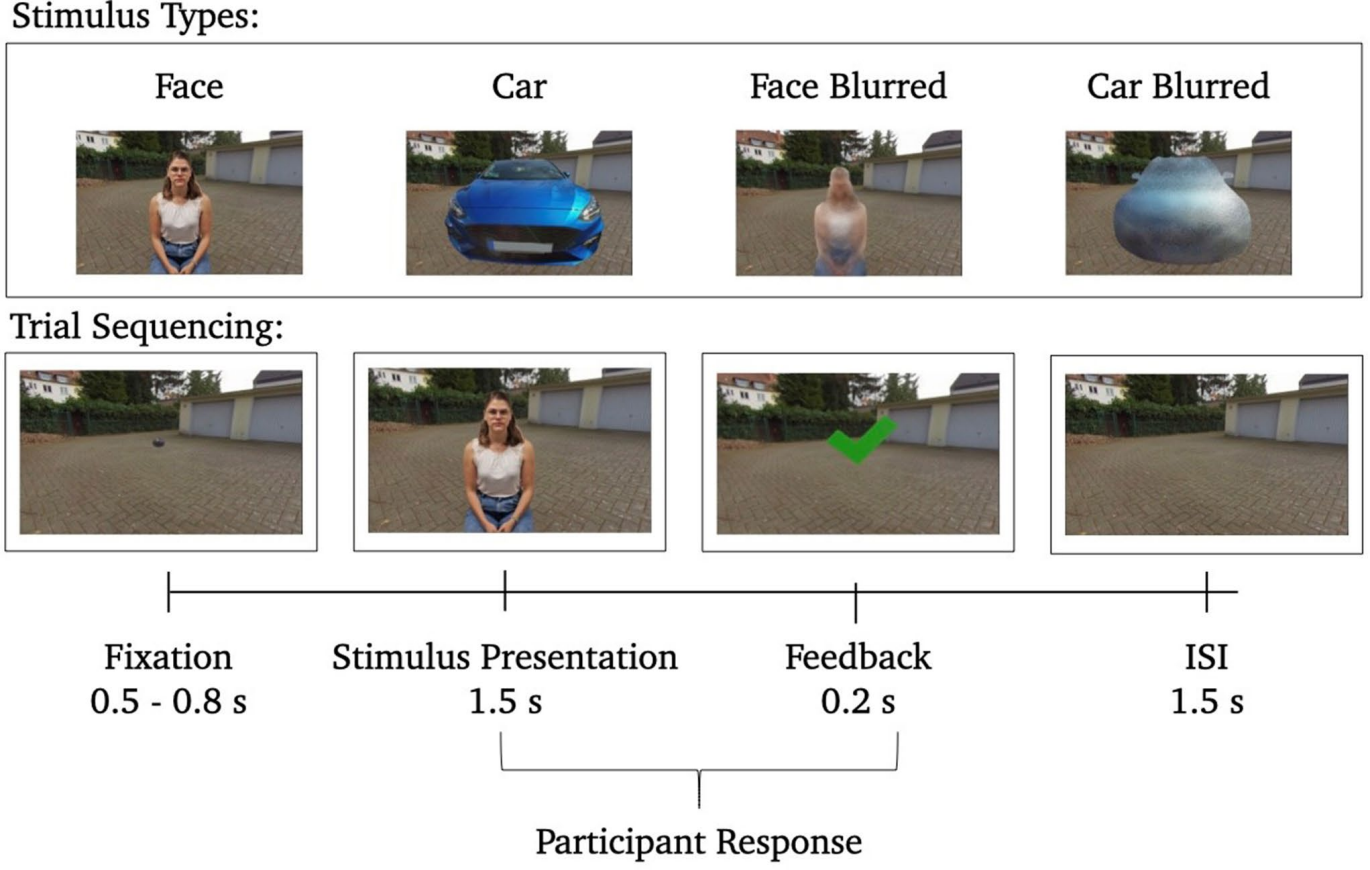
Stimulus material and trial sequence: 0.5–0.8 s fixation, 1.5 s stimulus presentation, 0.2 s feedback, 1.5 s inter stimulus interval (ISI). Examples of (blurred) face and (blurred) car stimuli are illustrated

The stimulus material encompassed a collection of 120 images featuring standard cars and 120 images portraying individuals seated on a stool, all set against the backdrop of an empty garage yard. These images were presented in both, 2D and 3D-360° formats. All individuals depicted in the images displayed neutral facial expressions, and any brand information or license plates were removed from the vehicles on display. All the images were captured using the Insta360 Pro VR-camera, offering a resolution of 8 K. Additionally, both cars and individuals were presented as blurred versions, which served as a standard control stimulus in the context of face perception (Bombari et al. 2013; Kuefner et al. 2010; Rossion 2014; Schwaninger et al. 2009). These blurred versions retained color information and perceptual form, while stimulus size and shape remained unaltered to intentionally reduce the semantic meaning (see Fig. 1).

### Procedure

All participants completed two conditions with the order of the PC and VR condition alternating among participants. Both experiments were conducted within a soundproof and electrically shielded room optimized for EEG measurements. Given the susceptibility of EEG data to motion-induced artifacts, participants were instructed to minimize movement and avoid looking around during the experiment, particularly in the VR environment. A five-minute break was provided between conditions during which the quality of the EEG signal was checked.

In the PC condition, participants were positioned in front of a standard PC monitor (24”, 1920 × 1200 resolution), maintaining a consistent distance of 115 cm from the screen. This setup resulted in a horizontal viewing angle of 7.5° and a vertical viewing angle of 5°. The 2D images were presented at the center of the screen using the Unity 5 video game engine (Version 2020).

In the VR condition, participants remained seated and wore a VR headset (HTC Vive Pro 2, 2448 × 2448 pixels per eye, offering up to a 120° field of vision and a 120 Hz refresh rate). The images were presented in 3D-360° at real-life scale, positioned at a distance of 62 cm for persons (with a horizontal viewing angle of 42° and vertical viewing angle of 98°) and 92 cm for cars (horizontal viewing angle of 166° and vertical viewing angle of 168°), thus maintaining the naturalistic proportions between the stimuli. Unity 5 was also utilized for stimulus presentation in the VR condition.

Randomized across participants, the stimuli from each category (faces and cars) were randomly allocated to two experimental conditions—PC or VR—resulting in 60 images of individuals and 60 images of cars per condition. It was ensured that no participant encountered the same person or car twice during the experiment. In total, each experimental condition encompassed 240 stimuli (60 normal persons, 60 blurred persons, 60 normal cars, and 60 blurred cars). The unaltered stimuli and their corresponding blurred control images were consistently presented within the same modality.

In both conditions, participants had to perform a standard stimulus discrimination task. Participants were asked to categorize all the presented stimuli into the categories of faces and cars by pressing the shoulder buttons of a USB gamepad with their index finger. Participants were asked to respond as quickly as possible and identify the category of the blurred images as either a face or a car as well. The side of the button press was alternated across participants (for even participant numbers the right button was pressed for “face” and the left button for “car”, for odd participant numbers the other way around).

Each of the 240 trials in every experimental condition adhered to a standardized sequencing structure, which was identical for both the PC and VR conditions (see Fig. 1). Following a fixation dot (0.5–0.8 s) that participants were asked to fixate at the beginning of every trial in both conditions, the images were presented for 1.5 s each and participants were expected to identify the stimulus category (face vs. car). This presentation was succeeded by a brief feedback lasting 0.2 s (i.e., green tick for correct answers, red cross for wrong answers), followed by an interstimulus interval (ISI) where a background image without a person or car was displayed for 1.5 s. Participants were instructed to blink or move exclusively during the ISI period to reduce ocular and movement artifacts. Consequently, each trial ranged from 3.7 to 4 s in duration, resulting in a total runtime of approximately eighteen minutes per experimental condition.

In both conditions, the trigger stream originating from Unity was transmitted to Lab Streaming Layer (LSL by SCCN, https://github.com/sccn/labstreaminglayer) to synchronize the EEG data stream and Unity triggers during measurement. The exact timing of the onset of stimulus triggers and the appearance of stimuli on the monitor or VR headset was previously validated using a photodiode. Furthermore, the time of button-press responses was recorded and saved as separate response-time files for each participant, subsequently integrated with the EEG data stream for further analysis (see Sect. 2.5).

### Behavioral data

The analysis of the response times was performed as part of the previous study analyzing the ERP. Detailed methodological descriptions and obtained results can also be found in the Statistical analysis and Results sections in Sagehorn et al. (2024). The response times from the behavioral discrimination task were averaged per stimulus type (face, blurred face, car, blurred car) across trials for each participant. The resulting average values were analyzed for differences between the stimulus types within each modality and between the two modalities in the same manner as the electrophysiological data (i.e., 2 × 4 rmANOVA; see Statistical Analysis).

### Electrophysiological recordings and preprocessing

Electrophysiological recordings (EEG) were obtained using 128 electrodes, adhering to the international 10–20 system, and were conducted throughout the entire experimental procedure encompassing both the PC and VR conditions. The Active-Two amplifier system from BioSemi (Amsterdam, Netherlands) was utilized with a sampling rate of 512 Hz and a bandwidth (3 dB) of 104 Hz. Additionally, a horizontal electrooculogram (hEOG) and a vertical electrooculogram (vEOG) were recorded, and reference and ground electrodes in the form of a common mode sense (CMS) and a driven right leg (DRL) electrode were employed.

The preprocessing of EEG data obtained from both modalities was uniformly conducted using MATLAB (Version R2022a, MathWorks Inc) and EEGLAB (Version 2024.0; Delorme and Makeig 2004) as well as inhouse scripts for wavelet analyses. In the initial step, the EEG data stream and trigger stream were integrated via the EEGLAB add-on MoBi-Lab (Ojeda et al. 2014). The data were then re-referenced to an average reference, high-pass filtered at 0.25 Hz, and low-pass filtered at 25 Hz. An automated artifact removal add-on (ASR; Mullen et al. 2015) was employed for the identification of noisy channels, followed by corresponding channel interpolation. Channels with a signal deviating more than two standard deviations from the mean signal were additionally interpolated. Linear detrending was applied to all channels to eliminate potential drifts. Independent component analysis (ICA; Delorme et al. 2007) was utilized for rejection of muscle, eye, heart, line noise and channel noise artifacts.

In preparation for the oscillatory analysis, data were epochized individually for each condition per participant, defining a time window around trigger onset of -500 to 1500 ms, with a baseline of -300 to 0 ms before stimulus onset.

### Oscillatory responses in electrode space

Spectral changes in oscillatory activity were examined using a Morlet wavelet analysis with a wavelet width of twelve cycles, a technique that is documented in existing literature (e.g., Bertrand and Pantev 1994; Tallon-Baudry and Bertrand 1999). A total of 39 wavelets spanning a frequency range from 2 Hz to 40 Hz was computed with a frequency resolution of 1. This process facilitates the creation of a time-by-frequency (TF) representation of the data, providing a dynamic measure of signal magnitude within each frequency band over time. Typically, induced oscillatory activity can be obscured in the averaged evoked potential due to latency variations. To preserve the signal of interest (i.e., the induced response), the TF amplitude was averaged in the frequency domain, i.e., across individual trial frequency transformations. This method enables the analysis of non-phase-locked components. Additionally, prior to the frequency decomposition, the evoked response (i.e., the ERP) was subtracted from each trial. This step was taken to allow for analyzing the non-phase-locked components of the signal as described in more detail elsewhere (see Cohen 2014; for similar procedure see e.g., Busch et al. 2006; Gruber et al. 2006).

**Fig. 2:**
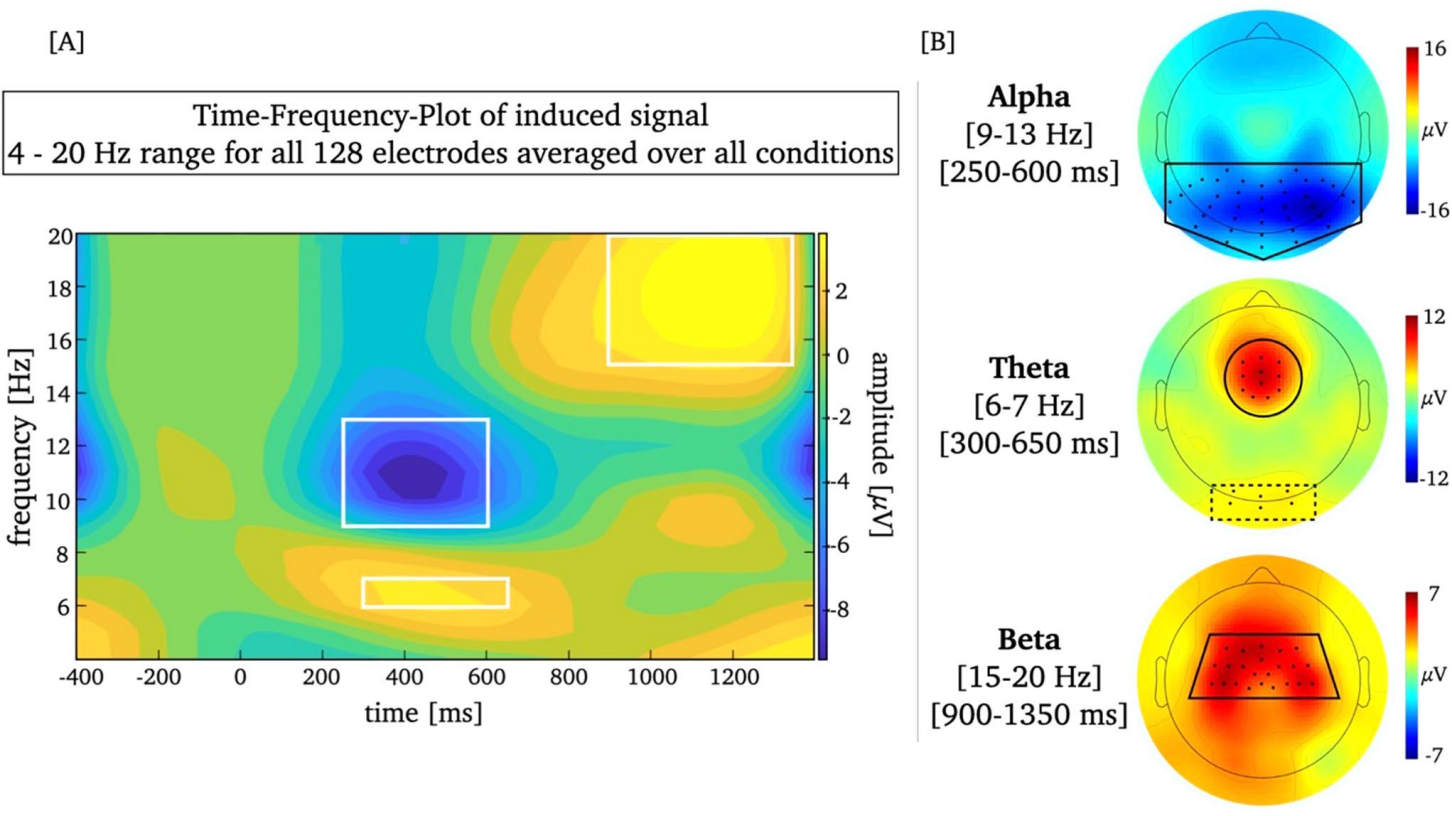
Panel A depicts the Time-Frequency (TF) plot of the induced oscillatory signal averaged over all conditions and all electrodes. Frequency ranges and time windows for the frequencies of interest are marked by a white rectangle. Panel B depicts the topographic distribution of the iABR, iTBR and iBBR averaged over all conditions. Electrodes selected for analyses are marked by black dots

The selection of relevant electrodes and time windows for further analyses was based on previous literature and adapted based on visual inspection of the average TF-plot (see Fig. 2A) and mean topographic distribution (see Fig. 2B) (for similar procedure see e.g., Gruber et al. 2006; Kisker et al. 2024). Based on visual inspection of the single-subject topographies of the frequencies of interest, two to five channels that were not identified by ASR were spherically spline-interpolated in eight participants.

Ultimately, the iABR was analyzed by means of the 9–13 Hz range at midline and temporal posterior electrodes (i.e., Oz, O1, O2, POz, PO3, PO4, PO7, PO8, Pz, P3, P4, P5, P6, P7, P8, P9, P10 and 21 surrounding electrodes; e.g., Kisker et al. 2021a; Klimesch et al. 1998) in the time window from 250 to 600ms. The iTBR was analyzed by means of the 6–7 Hz range at midfrontal electrodes (i.e., Fz, FCz, F1, F2 and seven surrounding electrodes; e.g., Dan and Reiner 2017; Puszta 2022) in the time window from 300 to 650ms. The iBBR was analyzed by means of the 15–20 Hz range including a centro-parietal electrode cluster (i.e., Cz, C1, C2, C3, C4, Fz, FCz, FC1, FC2, FC3, FC4 and 12 surrounding electrodes; e.g., Cardellicchio et al. 2020; Cassim et al. 2001) in the time window from 900 to 1350ms, i.e., during the post-response time window (see Behavioral data).

The visual inspection of the topographic distributions at the individual condition level (see Fig. 4) indicated an additional potentially relevant posterior electrode cluster for the iTBR that did not appear in the grand mean (electrodes marked in Fig. 2B). Therefore, the iTBR was analyzed exploratively at posterior electrodes (i.e., Oz, O1, O2 and three neighbouring electrodes; e.g., Kisker et al. 2024; Tang et al. 2022) in the same frequency range and time window as the midfrontal iTBR.

### Statistical analysis

The statistical analyses were performed using SPSS Statistics (IBM, Version 28). The response times from the discrimination task, the iABR, the iTBR, and the iBBR were analyzed as dependent variables using a 2 × 4 rmANOVA with the within-subject factors “modality” (VR vs. PC) and “stimulus type” (face vs. face-blurred vs. car vs. car-blurred). Whenever necessary, Greenhouse-Geisser-corrected *p*-values are reported.

Significant effects of the rmANOVA were complemented by post-hoc two-tailed paired samples *t*-tests. Effect sizes were calculated for all analyses (partial eta squared (*η*^2^) for the rmANOVA and Cohen’s *d* for post-hoc *t*-tests).

Based on the inspection of condition-wise topographic distributions, the iTBR was additionally analyzed at posterior electrodes in an exploratory rmANOVA, complementary to the analyses above.

### Oscillatory response in source space

**Fig. 3:**
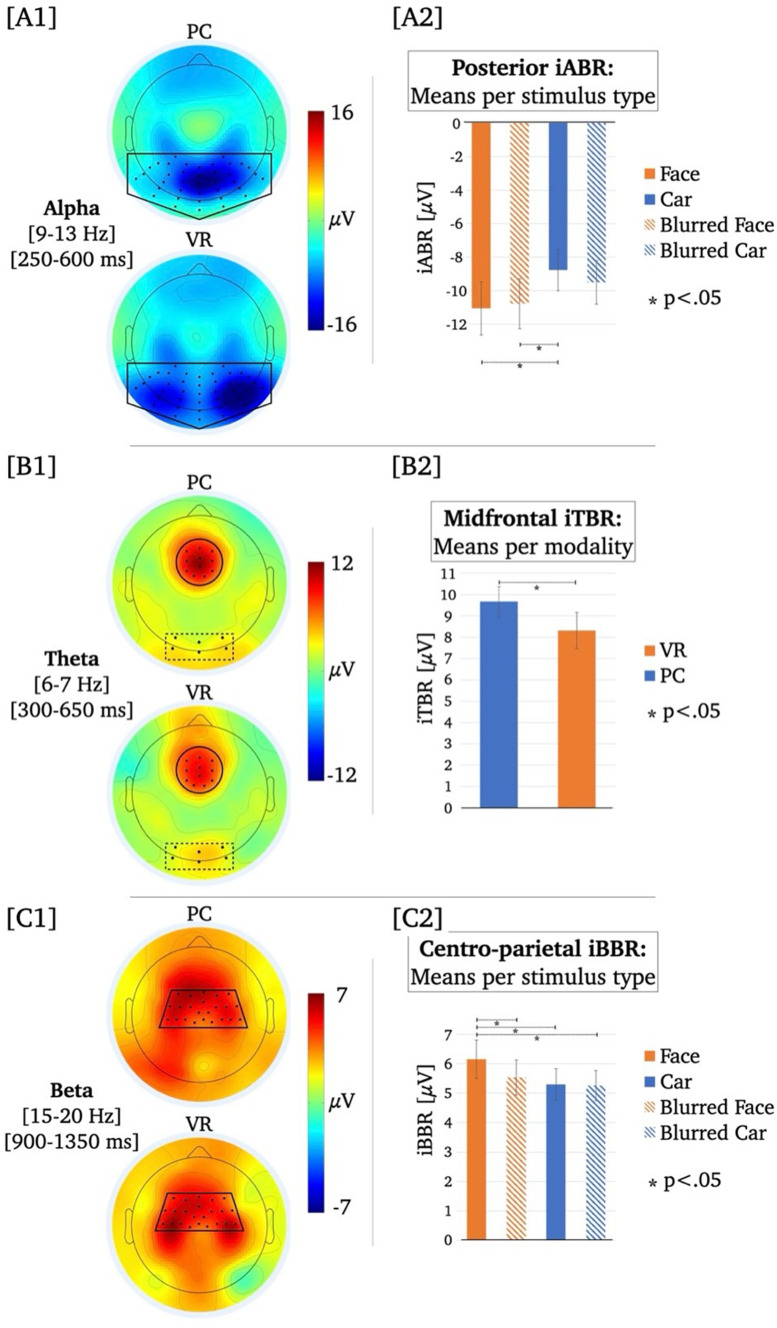
Panels A1, B1 and C1 depict the topographic distribution of the iABR, iTBR and iBBR per modality averaged over stimulus types. Electrodes selected for analyses are marked by black dots and black lines. Panels A2 and C2 depict bar charts illustrating mean differences in the posterior iABR and the mean centro-parietal iBBR per stimulus type averaged over modalities. Panel B1 depicts a bar chart illustrating mean differences in the midfrontal iTBR per modality averaged over stimulus types

**Fig. 4:**
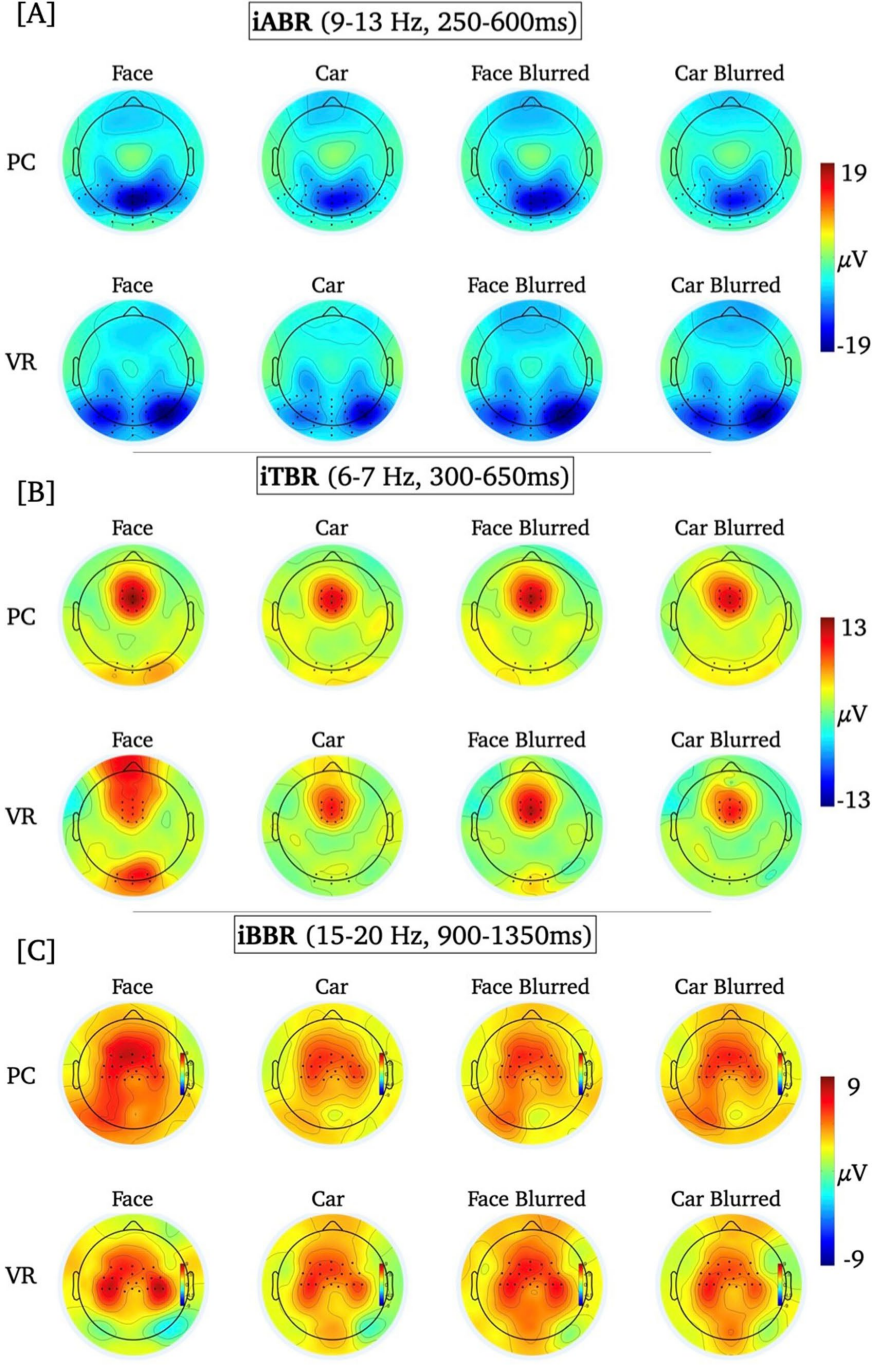
Topographic distributions for the iABR [**A**], the iTBR [**B**] and the iBBR [**C**] are given for all stimulus types in each modality separately

Concurrent to the analysis of the oscillatory responses in electrode space, comparisons that were of particular interest for the research question were additionally analyzed in source space. Thus, to further characterize the observed differences in iTBR between modalities (see Results and Fig. 3B), the prominent lateralization of iABR only under realistic VR conditions (see Fig. 3A1 and Fig. 4), and based on previous modality-specific source localization of ERP components (Sagehorn et al. 2023), we investigated the activation of the underlying cortical generators of the iTBR and iABR to determine whether they are distributed differently. We applied variable resolution electromagnetic tomography (VARETA; Bosch-Bayard et al. 2001), which provides an intracranial distribution of current densities in the source space that is spatially smoothest and highly compatible with the amplitude distribution in the electrode space. An inverse solution consisting of 3244 grid points was defined by a leadfield matrix and corresponded to the placement of the 128-channel EEG system (10–20 system).

Using Hotelling’s T^2^ test for each effect of interest (i.e., PC-iABR vs. VR-iABR, PC-iTBR vs. VR-iTBR), activation patterns were tested against zero to localize respective cortical sources. To validate the robustness of the localization of oscillatory responses using VARETA, the sources of the mean iABR and mean iTBR per condition were first localized, respectively. After confirming consistency with previous publications (Cavanagh and Frank 2014; Ishii et al. 1999; Jensen and Mazaheri 2010; Köster and Gruber 2022; Manshanden et al. 2002), the effects of interest were investigated. For these differences between modalities, the significance level was set to *p* < .05 and the critical *t*-value was *t*_crit_ = 40.1. Significant voxels were projected onto the cortical surface which was constructed on the basis of the average probabilistic MRI brain atlas by the Montreal Neurological Institute (MNI; Evans et al. 1993) (See Fig. 4).

## Results

### Behavioral data

The detailed descriptive and inferential statistics for the behavioral data analysis are reported in Sagehorn et al. (2024) and can be found in Tables S3 and S4, Supplementary Material. In summary, a significant main effect for the factor “modality” (*F*_modality_(1, 57) = 222.43, *p* < .001, *η*^*2*^ = 0.90) revealed that the response times for the same stimulus type (e.g., PC face vs. VR face) were consistently faster in the VR modality than in the PC modality (*M*_PC_ = 549.9 ms; *M*_VR_ = 535.7 ms). In addition, the main significant effect for the factor “stimulus type” (*F*_*stimulus*_(2.1, 120.1) = 41.28, *p* < .001, *η*^*2*^ = 0.42) showed that faces elicited the slowest reaction times compared to all other stimuli in both modalities (Face: *M*_PC_ = 559.4 ms; *M*_VR_ = 550.2 ms). The significant interaction effect (*F*_*interaction*_(2.4, 136.2) = 9.10, *p* < .001, *η*^*2*^ = 0.14) moreover revealed that in VR, participants reacted fastest to cars (*M* = 524.6 ms) and in the PC modality the fastest response times occurred with cars (*M* = 544.4 ms) and blurred cars (*M* = 543.7 ms).

### iABR

The rmANOVA for the iABR revealed no significant main effect for the factor “modality” (*F*_modality_(1, 54) = 2.45, *p* = .124), a significant main effect for the factor “stimulus type” (*F*_*stimulus*_(2.6, 138.2) = 5.27, *p* = .003, *η*^*2*^ = 0.09), and no significant interaction effect (*F*_*interaction*_(3, 162) = 1.80, *p* = .250). The respective descriptive statistics are given in Table S5, Supplementary Material. Irrespective of modality, the iABR was more negative, i.e., there was stronger alpha suppression, for face stimuli than car stimuli and for car stimuli than blurred faces (see Table 1; Figs. 3A and 4).

**Table 1:** Pairwise comparison of main factor “Stimulus Type” (averaged over modalities) for the iABR and the iBBR

	df	T	*p*	Cohen´s d
**iABR**				
Face vs. Car	54	-3.15	0.003**	− 0.43
Face vs. Face-B	54	− 0.46	0.646	− 0.06
Face vs. Car-B	54	-1.98	0.052	− 0.27
Car vs. Face-B	54	3.20	0.002**	0.43
Car vs. Car-B	54	1.30	0.199	0.18
Face-B vs. Car-B	54	-1.00	0.051	− 0.27
**iBBR**				
Face vs. Car	54	2.32	0.024*	0.31
Face vs. Face-B	54	2.24	0.029*	0.30
Face vs. Car-B	54	2.36	0.022*	0.32
Car vs. Face-B	54	− 0.68	0.499	− 0.10
Car vs. Car-B	54	0.15	0.880	0.02
Face-B vs. Car-B	54	0.88	0.384	0.12

*Notes* *significant for α = 0.05, **significant after Bonferroni-correction

**Fig. 5:**
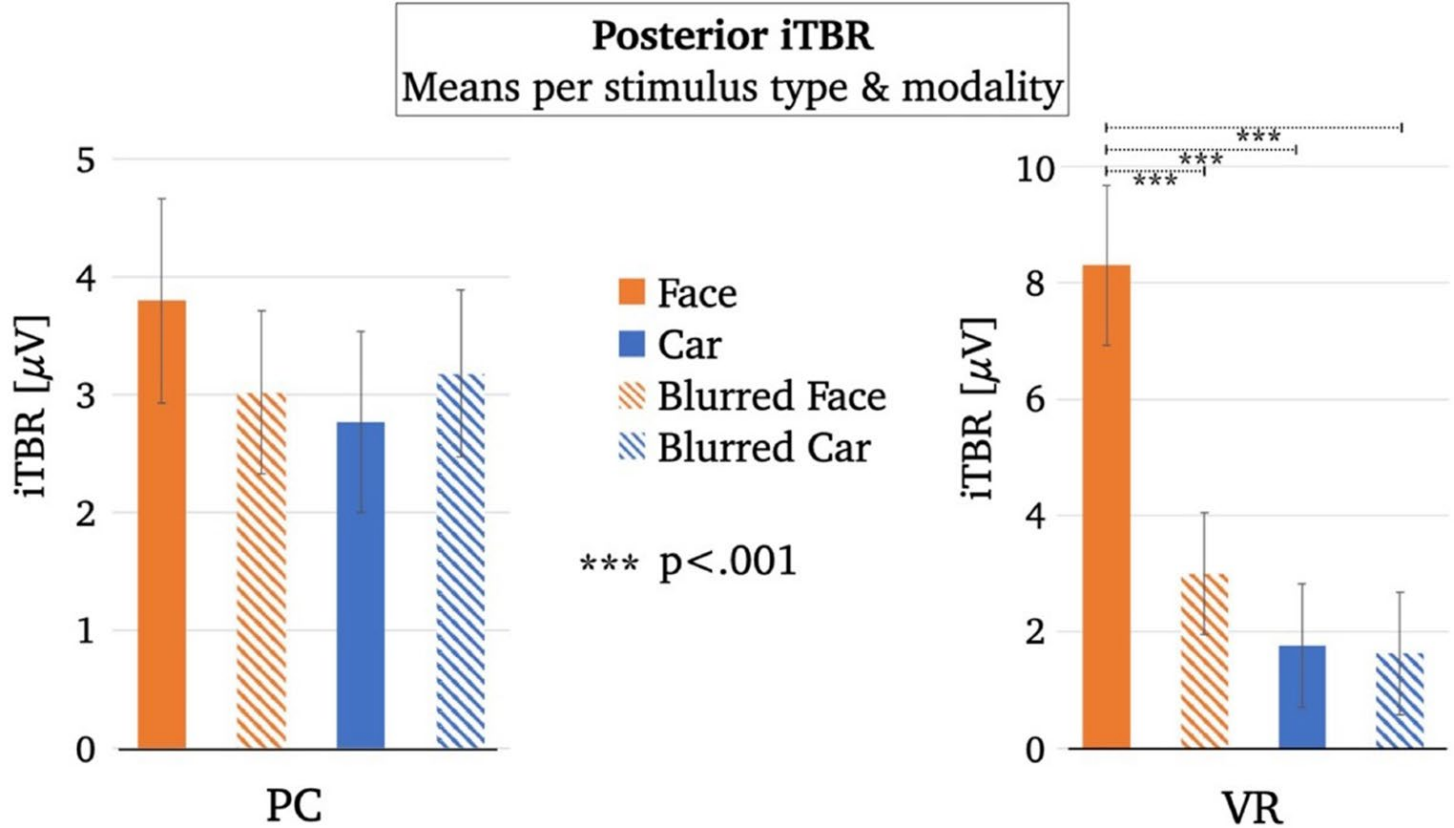
Bar charts illustrating mean differences in the posterior iTBR per stimulus type and modality

#### iABR in source space

**Fig. 6:**
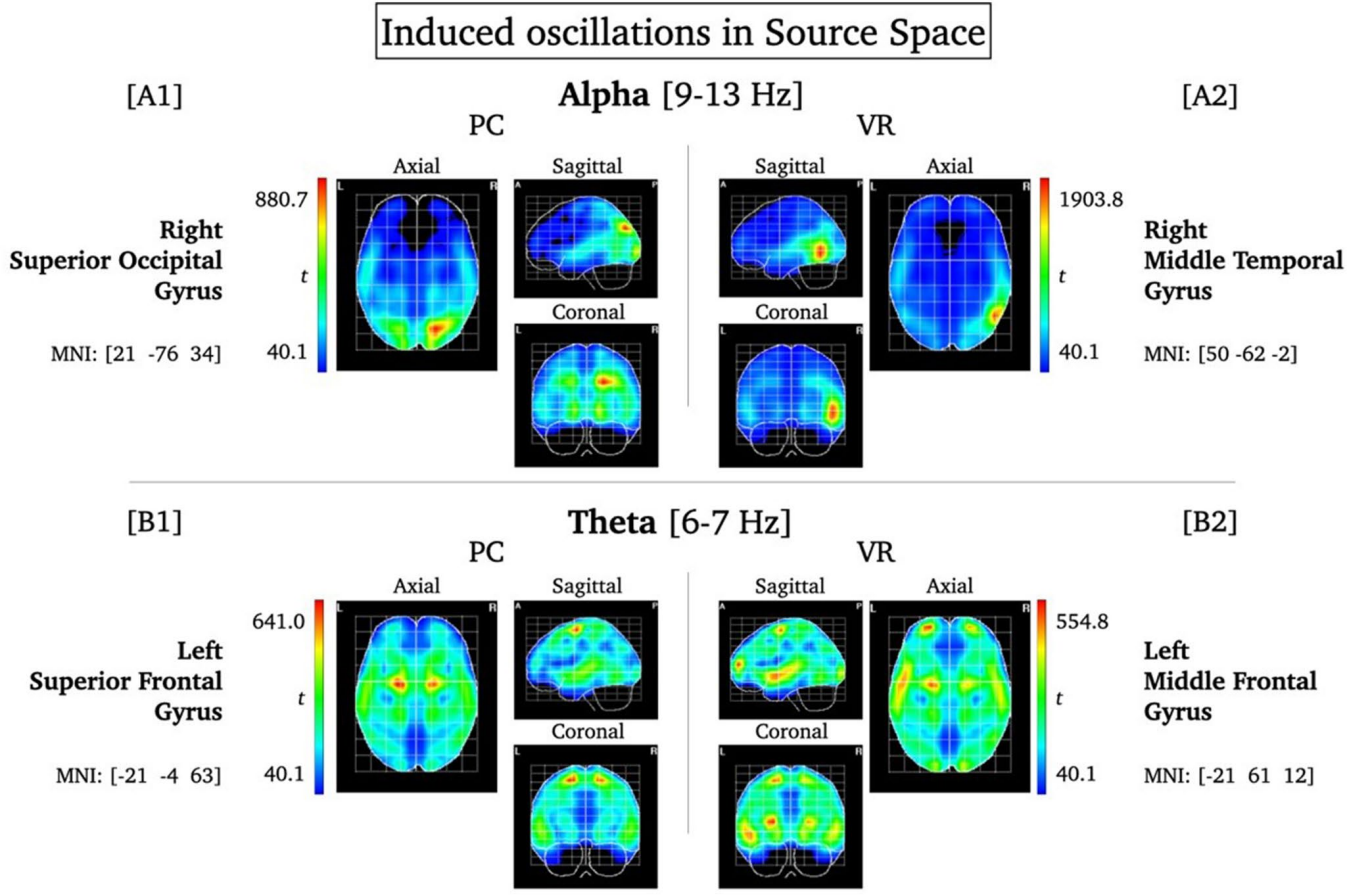
Projections of statistically significant sources of the mean iABR (9–13 Hz; 250–600 ms) and the mean iTBR (6–7 Hz; 300–650 ms) across stimulus types and per modality tested against zero with *p* < .05, *t*_crit_ = 40.1. Per panel, the center of gravity is labeled and the respective MNI coordinates are given

The iABR averaged over all stimulus types under laboratory conditions was localized in the right superior occipital gyrus (see Fig. 6A1), which is consistent with previous literature (Jensen and Mazaheri 2010; Köster and Gruber 2022; Manshanden et al. 2002). The iABR averaged over all stimulus types under realistic virtual conditions was localized in the right middle temporal gyrus (see Fig. 6A2).

**Fig. 7:**
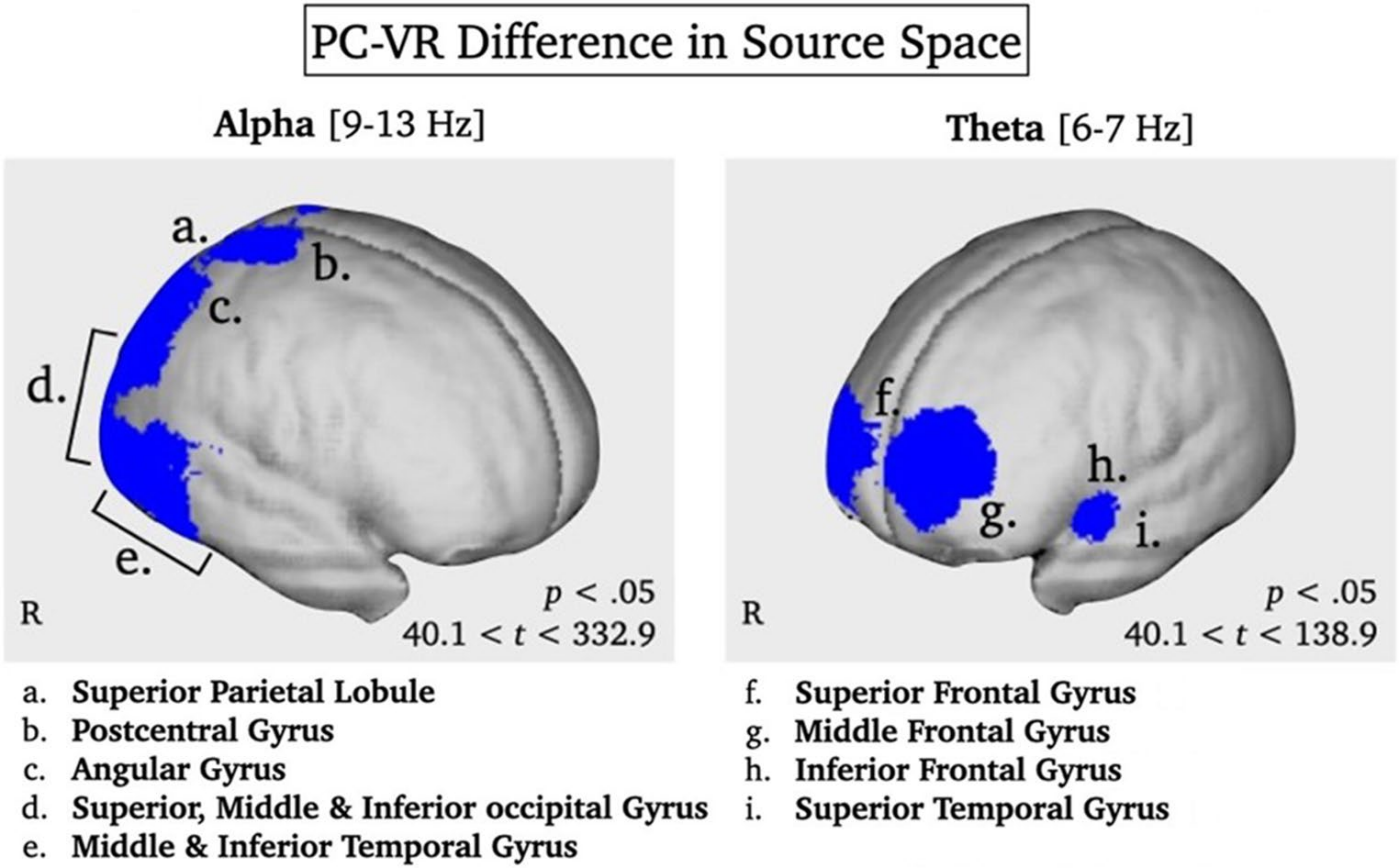
Statistically significant sources of the PC-VR difference of the mean iABR (9–13 Hz; 250–600 ms) and the mean iTBR (6–7 Hz; 300–650 ms) across stimulus types projected onto the surface of an average MRI template brain. Statistically significant differences in activity between modalities are marked in blue (*p* < .05, *t*_crit_ = 40.1)

The difference of iABR between PC and VR yielded significantly different activity in the bilateral superior occipital gyrus with the center of gravity on the right, accompanied by the lingual gyrus, occipital pole, and cuneus as well as superior parietal lobule and the post-central gyrus (all bilateral). Further significant differences were found in the right superior, middle and inferior temporal gyrus, the right middle and inferior occipital gyrus, the right angular gyrus and the right lateral occipitotemporal gyrus (see Fig. 7).

### iTBR

The rmANOVA for the midfrontal iTBR revealed a significant main effect for the factor “modality” (*F*_*modality*_(1, 54) = 4.64, *p* = .036, *η*^*2*^ = 0.08), no significant main effect for the factor “stimulus type” (*F*_*stimulus*_(3, 162) = 2.58, *p* = .057), and no significant interaction effect (*F*_*interaction*_(3, 162) = 1.47, *p* = .224). The respective descriptive statistics are given in Table S5, Supplementary Material. The midfrontal iTBR averaged over all stimulus-types was stronger in the PC condition than the VR condition (*M*_PC_ = 9.66µV, *M*_VR_ = 8.31µV; see Fig. 3B).

The explorative rmANOVA for the posterior iTBR revealed no significant main effect for the factor “modality” (*F*_*modality*_(1, 54) = 0.44, *p* = .511), a significant main effect for the factor “stimulus type” (*F*_*stimulus*_(2.5, 132.5) = 10.86, *p* < .001, *η*^*2*^ = 0.17), and a significant interaction effect (*F*_*interaction*_(3, 162) = 6.97, *p* < .001, *η*^*2*^ = 0.11). The respective descriptive statistics are given in Table S5, Supplementary Material. Based on the significant interaction effect, post-hoc *t*-tests were performed between all stimulus types within each modality, respectively. Within the PC condition, the posterior iTBR did not significantly differ between the stimulus types (Table 2; Fig. 5). Within the VR condition, the posterior iTBR was stronger for faces than all other stimulus types (Table 2; Fig. 5). Comparing the posterior iTBR between modalities, it was significantly stronger for VR faces than PC faces (Table 2).

**Table 2:** Pairwise comparisons of the posterior iTBR within and between modalities

	df	T	*p*	Cohen´s d
**PC**				
Face vs. Car	54	1.05	0.297	0.14
Face vs. Face-B	54	0.97	0.336	0.13
Face vs. Car-B	54	0.57	0.571	0.08
Car vs. Face-B	54	− 0.29	0.772	− 0.04
Car vs. Car-B	54	− 0.47	0.640	− 0.06
Face-B vs. Car-B	54	− 0.18	0.857	− 0.02
**VR**				
Face vs. Car	54	5.16	< 0.001**	0.70
Face vs. Face-B	54	4.01	< 0.001**	0.54
Face vs. Car-B	54	5.00	< 0.001**	0.67
Car vs. Face-B	54	-1.12	0.266	− 0.15
Car vs. Car-B	54	0.14	0.893	0.02
Face-B vs. Car-B	54	1.22	0.230	0.16
**PC vs. VR**				
Face	54	-3.35	0.001**	− 0.45
Car	54	0.95	0.345	0.13
Face Blurred	54	0.02	0.984	0.00
Car Blurred	54	1.35	0.183	0.18

*Notes* *significant for α = 0.05, **significant after Bonferroni-correction

#### iTBR in source space

The iTBR averaged over all stimulus types in the PC condition was localized in the left superior frontal gyrus (see Fig. 6B1), which is in accordance with previous literature (Cavanagh and Frank 2014; Ishii et al. 1999). The iTBR averaged over all stimulus types under realistic virtual conditions was localized in the left middle frontal gyrus (see Fig. 6B2).

The difference of iTBR between PC and VR yielded significantly different activity in the bilateral middle frontal gyrus with the center of gravity in the left hemisphere, accompanied by the superior frontal gyrus, lingual gyrus, and occipital pole (all bilateral). Further significant differences were found in the left superior temporal gyrus, the left precentral gyrus, the left inferior frontal gyrus, the right superior occipital gyrus, and the right cuneus (see Fig. 7).

### iBBR

The rmANOVA for the iBBR revealed no significant main effect for the factor “modality” (*F*_modality_(1, 54) = 0.142, *p* = .707), a significant main effect for the factor “stimulus type” (*F*_*stimulus*_(2.2, 117.9) = 3.25, *p* = .038, *η*^*2*^ = 0.06), and no significant interaction effect (*F*_*interaction*_(2.0, 110.7) = 1.80, *p* = .169). The respective descriptive statistics are given in Table S5, Supplementary Material. Irrespective of modality, iBBR was more positive, i.e., there was a stronger post-movement beta band rebound, for faces than for all other stimulus types (see Table 1; Fig. 3C).

## Discussion

### Summary

The aim of the present study was to extend and complement previous research on the neural mechanisms of processing realistic human faces and objects with novel findings from the frequency domain by comparing a 2D laboratory approach with a 3D VR environment (Sagehorn et al. 2023, 2024). The induced oscillatory responses provide information about the neural dynamics that are elicited by but not strictly phase-locked to the presented stimuli. In the laboratory environment, participants saw 2D images on a PC monitor; in the VR environment, they saw life-size 3D representations. In both conditions, the participants actively differentiated between stimulus categories (faces vs. cars) by pressing a button.

The results provide further insight into how the basic neural mechanisms involved in the perception and processing of faces and objects are affected by different degrees of realism. Specifically, potential modality-dependent differences in attention, cognitive load, and task-related post-movement processing were investigated by comparing the induced oscillatory responses in the alpha, theta and beta bands, respectively, providing insights into less strictly phase-locked processing mechanisms than the previously investigated neural correlates (Kirasirova et al. 2021; Sagehorn et al. 2023, 2024; Stolz et al. 2019). No evidence was found for differences in stimulus-dependent attention or task-related post-movement processing between 2D laboratory conditions and realistic virtual conditions, as posterior alpha suppression and re-synchronization of centro-parietal beta did not differ between conditions, only between stimulus types. Faces generally elicited relatively higher attentional and task-related post-movement processing compared to car stimuli in both conditions. Yet, differences in the recruited attentional networks underlying the iABR suggest more stimulus-specific processing under realistic virtual conditions. Cognitive load was higher in the 2D setting, as midfrontal theta was significantly stronger under laboratory conditions. In contrast, exploratory analysis of posterior theta showed stronger responses in VR, especially for faces. Moreover, the iTBR originated from different cortical sources depending on the modality, suggesting enhanced semantic processing under realistic virtual conditions.

### Attentional demand in realistic virtual settings

The analysis of the posterior iABR revealed no significant effect of modality on attention, and only differed between stimulus types in both conditions. At first, this result seems surprising, as a higher attentional demand would have been expected under realistic virtual conditions due to the larger stimulus size and proximity. Several previous studies found increased explicit attention in VR compared to 2D (e.g., Kober et al. 2012; Makransky et al. 2019; Schubring et al. 2020), however, this is not supported by the present results. Others found similar alpha band responses between 2D and 3D for simple tasks, and only encountered differences in more complex tasks (Dan and Reiner 2017). In the present study, the similar amount of attention required could indicate that the orientation of the attentional focus on the relevant stimulus is comparable in both conditions. Thus, it is possible to implement a simple paradigm such as distinguishing between faces and cars in a realistic virtual setting without immensely increasing the attentional demand and thus possibly masking other effects. Differences in the neuronal response to faces and other objects in VR compared to images on a PC, as already found in other studies in the ERP domain (e.g., Kirasirova et al. 2021; Sagehorn et al. 2023, 2024; Stolz et al. 2019), therefore cannot be attributed solely to an increased attentional demand in VR environments but to characteristics inherent to the stimuli themselves.

Even though a similar iABR suggests comparable attentional demands when comparing laboratory and realistic virtual conditions, the source analysis revealed differences in the neural generators underlying these responses. The iABR under realistic virtual conditions appears to originate from areas located further along the visual perceptual pathway and specialized for face and object recognition and semantic memory (Bonner and Price 2013; Brambati et al. 2010; Farahibozorg et al. 2022; Wang et al. 2018), suggesting more stimulus-specific processing than under laboratory conditions. In particular, the source differences between the two modalities lie not only in general visual processing areas but also affect parts of the extended face processing network (Wang et al. 2018). This is consistent with other findings indicating network-level differences between 2D and 3D perception based on stronger functional connectivity within the occipital and parietal networks underlying alpha band activity (Xu and Sui 2021). Thus, although they ultimately require comparable attentional resources, the neural network activation differs depending on the modality.

Consistent with the inherent relevance of faces, the significant effect of stimulus type on the iABR across modalities indicates a higher attentional demand for face stimuli compared to car stimuli. It has been shown that alpha band activity interacts with lower frequency bands during emotional processing and that this activity is higher for emotional stimulus material than for neutral material (Aktürk et al. 2020; Codispoti et al. 2023; Güntekin and Başar 2014). Given their personal relevance and importance in everyday interactions, faces could be considered to have a stronger emotional connotation than cars, even without explicitly showing an emotion in the sense of an emotional facial expression. The increased attentional demand for faces compared to cars could therefore be due to their inherent self-referential value for the observer. Considering the higher emotional and self-referential value of faces, additional processing steps might be required that cause slower responses than cars as less inherently significant stimuli. This could also explain why slower reaction times were observed for faces compared to cars in both modalities, although other results have often been reported in previous literature (Dering et al. 2009; Kuefner et al. 2010). Given the higher emotional and self-referential value of faces, additional processing steps may be required, leading to slower responses than for cars as less demanding stimuli.

Surprisingly, there were no significant differences between the iABR for the sharp and the blurred images of the same stimulus category. Since the blurred images have less semantic meaning and are only equivalent in terms of basic perceptual features, it would have been expected that there would also be differences in attention compared to the sharp images. One possible explanation could be that although the blurred images were supposed to be reduced in semantic meaning, they were still strongly associated with the respective stimulus category. The silhouette of the stimulus could therefore be sufficient as a conceptual cue to trigger a semantic value comparable to that of the sharp images. In addition, participants were also asked to assign the blurred images to their respective stimulus category (face vs. car), which may have further strengthened the semantic association of the blurred images. To further investigate whether the attentional demand for faces and cars differs from less semantically charged stimuli of similar perceptual quality, adapted perceptual controls are necessary and the behavioral task would have to be optimized so that a specific response can also be given for the blurred controls.

### Cognitive load and depth perception in realistic virtual conditions

The analysis of the midfrontal iTBR showed that cognitive load demands differed between modalities. Consistent with previous research comparing cognitive load between 2D and 3D environments (Dan and Reiner 2017), the iTBR was stronger in the laboratory condition than in the realistic virtual condition, suggesting a higher processing demand for the 2D stimuli than the 3D stimuli. Considering that the virtual setting provided participants with an environment in which they encountered the stimuli to be differentiated in their natural proportions and in physical proximity to themselves, stimulus identification is more natural and therefore easier to perform. This is also reflected in the behavioral responses which show faster reaction times overall in the VR condition (see also Sagehorn et al. 2024).

In contrast, other studies showed cognitive load to be higher in 3D than in 2D environments for various tasks (Li et al. 2020; Makransky et al. 2019; Slobounov et al. 2015; Xu and Sui 2021). These ranged from passive viewing of dynamic scenes (Xu and Sui 2021) to spatial navigation (Slobounov et al. 2015) or performing multiple tasks within a simulated virtual environment (Makransky et al. 2019). Moreover, the operationalization of cognitive load is very inconsistent across studies. While cognitive load can be indexed by the iTBR (Dan and Reiner 2017), other investigate the evoked theta signal to access more stimulus-specific processing (Li et al. 2020) or combine responses from different frequency bands as a more extensive metric (Makransky et al. 2019). Ultimately, several factors interact to contribute to the ambiguity of results regarding cognitive load, most notably the degree of realism of the different types of virtual environments (i.e., photorealistic images, programmed simulations, mixed designs, etc.), the task itself (e.g., learning versus stimulus discrimination), and the operationalization of the target process (i.e., evoked or induced signal; time, frequency, or source domain; combination of signals). In any case, depending on the mechanisms to be studied, the neural correlates to be investigated and the tasks to be performed, the use of VR settings may not always be the superior choice. However, based on the present results showing a reduction in cognitive load for the realistic 3D stimuli, VR seems to be a valuable tool for perception research.

In addition to the midfrontal iTBR, the data indicated a possible difference in the posterior iTBR between modalities and stimulus types, so an additional exploratory analysis of the iTBR was performed on a posterior electrode cluster. The results showed a differentiated posterior iTBR for the realistic virtual conditions, in particular for faces. The virtual face stimuli elicited a stronger iTBR than all other 3D stimuli and 2D faces. Thus, the complexity of faces in realistic virtual conditions, where they are presented in real proportions and in close spatial proximity to the participant, appears to require differentiated processing that is not apparent for the other stimuli. Similar results have already been shown in studies comparing the visual perception of objects in 2D and 3D (Kisker et al. 2024; Tang et al. 2022), suggesting that the posterior iTBR may be specifically involved in the perception of complex three-dimensional stimuli with depth information that is not transmitted in 2D settings. Given the previously observed involvement of the parieto-occipital theta in the processing of faces (Dravida et al. 2019), the specific response pattern under realistic virtual conditions clearly requires a more explicit targeting of the posterior iTBR as a potential marker to discriminate 2D and 3D perception, possibly even specifically for faces. A reliable neural marker for social cognition would also be useful in diagnostic and training scenarios, as previous electrophysiological markers from laboratory studies have failed to accurately reflect behavioral improvements in social functioning (Key and Corbett 2020). So far, the application of VR in training social skills, cognition and functioning seems very promising (Kandalaft et al. 2013; Parsons and Mitchell 2002), which could be further supported by evidence from neurophysiological assessments.

Further supporting the notion that the realistic 3D stimuli require adapted processing, source analysis revealed that the iTBR under realistic virtual conditions seems to involve social cognition (Martín-Luengo et al. 2023; Molenberghs et al. 2016) and additional semantic memory processing that might provide access to stimulus specific information (Bonner and Price 2013; Brambati et al. 2010; Farahibozorg et al. 2022), whereas the iTBR under laboratory conditions is rather strictly related to working memory processes (Boisgueheneuc et al. 2006). Moreover, the source differences between PC and VR conditions are located in areas of the extended face processing and mentalizing networks (Wang et al. 2018), also suggesting that the perception of 2D monitor and 3D VR stimuli require distinct network activations, respectively. Taken together with the results from the ERP domain (Sagehorn et al. 2024) indicating deeper conceptual processing of the stimuli under realistic virtual conditions, these results support the potential of realistic stimulus presentation using VR to gain more comprehensive insights into neural processing, especially in the area of visual perception.

### Task-related post-movement processing in realistic virtual settings

Like the iABR, the analysis of centro-parietal resynchronization of the iBBR showed only an effect of stimulus type but not of modality on task-related post-movement processing. The response to faces elicited stronger beta band resynchronization after the button press than any other stimulus type in both conditions. At this point, we can only speculate as to why the response pattern for faces is particularly different from the other stimulus types, as the response times do not indicate faster reactions to faces than cars, but rather the opposite (see Sagehorn et al. 2024). While a decrease in the iBBR during the response is directly related to movement execution, the rebound of iBBR is also associated with complex integrative processes that go beyond motor functions (Cardellicchio et al. 2020). Taking that into consideration, the processing of faces might require additional resources that are not triggered by blurred faces or car stimuli. Whether this is the case, and exactly which resources are used here is beyond the scope of the present study.

However, since we found no differences in post-movement processing for the VR setting and wearing the HMD, the transfer of conventional paradigms in which participants perform simple response tasks is feasible, so that effects such as the face-specific iBBR could also be further investigated under realistic virtual conditions.

### Implications for perceptual paradigms in VR

Taken together, modality-specific perceptual processing of faces and objects cannot be explained by varying demands in attention and cognitive load alone. Both the previous results from the ERP domain (Sagehorn et al. 2023, 2024) and the present results from the frequency domain indicate more in-depth processing of realistic stimuli in VR that requires additional access of semantic information and social cognition. With the aim of gaining a deeper understanding of the perception of semantically relevant stimuli such as faces or everyday objects, VR represents a powerful tool for investigating the underlying neural mechanisms under consideration of social relevance, emotional, and decisional factors. However, contrary results coming from studies investigating other cognitive processes (e.g., learning; Johnsdorf et al. 2023b; Schubring et al. 2020) and applying different forms of virtual environments that vary in perceived realism (e.g., simulated VR; Makransky et al. 2019), emphasize that the implementation of more realistic virtual conditions using VR should be considered carefully, as it may not always be effective and may even impede the target process. In fact, the implementation of 3D tasks in VR should not simply be seen as advanced analog versions of 2D tasks, but rather as an experimental approach in its own right. Within the field of visual perception, however, the use of realistic virtual environments offers the potential to approximate real-life relevant processing mechanisms by providing crucial stimulus properties such as relative size, depth information, and spatial proximity that are lacking in conventional 2D laboratory environments.

In any case, considering the increasing use of VR technology in recreational, educational and clinical contexts (Suh and Prophet 2018), it is important to investigate cognitive processing within VR settings from a neuropsychological perspective as well. VR setups are nowadays affordable and can easily be used at home, opening up versatile possibilities for VR gaming (Koivisto and Hamari 2019) and even daily activities (Lee et al. 2020; Xi and Hamari 2021). Similarly, it is becoming easier to use VR in education and learning (Marougkas et al. 2023; McGovern et al. 2020), and even in the clinical field, VR approaches offer promising applications for diagnostic, therapeutic and rehabilitative purposes (Clus et al. 2018; Glennon et al. 2018; Gonzalez-Argote 2022). To weigh the risks and benefits of all these applications of VR in terms of impact on cognitive processing, psychological and neurophysiological research should continue to develop experimental setups in VR.

## Conclusion

In the present study we were able to show that the transfer of a standard face perception paradigm to a more realistic virtual setting does not increase the attentional demands, and even decreases the cognitive load, which might be explained by the more natural perceptual characteristics and congruent context embedding. The neural sources involved in stimulus processing under realistic virtual conditions suggest additional semantic information processing specific to the stimulus material and enhanced social cognition, which is not evident under laboratory conditions, leading to modality-specific activations within the face and mentalization networks. These important findings lay the groundwork for more in-depth studies of oscillatory responses to real-life relevant stimuli such as faces and other objects and highlight the potential to extend existing knowledge of perceptual processes using more realistic stimulus presentation by means of VR. Going forward, advances in VR technology offer the possibility to further investigate the neural underpinnings of face processing under consideration of emotional and social aspects by manipulating facial expressions and creating dynamic virtual scenarios.

## Electronic supplementary material

Below is the link to the electronic supplementary material.

Supplementary Material 1

## Data Availability

The datasets generated during and analyzed during the current study are available from the corresponding author on reasonable request. The stimulus material used in this study is available upon reasonable request and may be used for scientific purposes only. Access under: https://www.psycho.uni-osnabrueck.de/fachgebiete/allgemeine_psychologie_i/luvre.html.
