# Supplementary material for: A comparative analysis of face and object perception in 2D laboratory and virtual reality settings: insights from induced oscillatory responses
Source: Exp Brain Res. 2024 Oct 12;242(12):2765–83. doi: 10.1007/s00221-024-06935-3 (PMC11568981; doi:10.1007/s00221-024-06935-3)
Supplement: Supplementary file 1 — Supplementary Material 1 [file 221_2024_6935_MOESM1_ESM.docx]

**Supplementary Material**

**Table S3**

*Descriptive statistics (mean, standard deviation, confidence interval) for the Response Times in ms for both modalities and all stimulus types*

|  | *M* | *SD* | Confidence interval | |
| --- | --- | --- | --- | --- |
|  |  |  | Lower limit | Upper limit |
| *PC* |  |  |  | |
| Face | 559.4 | 8.0 | 557.3 | 561.5 |
| Car | 544.4 | 8.7 | 542.0 | 546.7 |
| Face Blurred | 552.0 | 9.3 | 549.5 | 554.5 |
| Car Blurred | 543.7 | 9.7 | 541.1 | 546.3 |
| *VR* |  |  |  |  |
| Face | 550.2 | 15.6 | 546.1 | 554.4 |
| Car | 524.6 | 10.8 | 521.8 | 527.5 |
| Face Blurred | 541.0 | 11.7 | 537.9 | 544.1 |
| Car Blurred | 526.8 | 11.8 | 523.7 | 530.0 |

**Table S4**

*Pairwise comparison of the Response Times in ms within and between modalities*

|  | *df* | *T* | *p* | *Cohen´s d* |
| --- | --- | --- | --- | --- |
| P*C* |  |  |  |  |
| Face vs. Car | 57 | 21.19 | <.001** | 2.80 |
| Face vs. Face-B | 57 | 11.93 | <.001** | 1.57 |
| Face vs. Car-B | 57 | 22.57 | <.001** | 2.96 |
| Car vs. Face-B | 57 | -6.11 | <.001** | -.80 |
| Car vs. Car-B | 57 | 1.18 | .242 | .16 |
| Face-B vs. Car-B | 57 | 7.90 | <.001** | .90 |
| *VR* |  |  |  |  |
| Face vs. Car | 57 | 20.61 | <.001** | 2.71 |
| Face vs. Face-B | 57 | 9.41 | <.001** | 1.24 |
| Face vs. Car-B | 57 | 19.48 | <.001** | 2.56 |
| Car vs. Face-B | 57 | -35.76 | <.001** | -4.70 |
| Car vs. Car-B | 57 | -6.53 | <.001** | -.86 |
| Face-B vs. Car-B | 57 | 29.46 | <.001** | 3.87 |
| *PC vs. VR* |  |  |  |  |
| Face | 57 | 4.43 | <.001** | .58 |
| Car | 57 | 22.88 | <.001** | 3.01 |
| Face Blurred | 57 | 6.29 | <.001** | .83 |
| Car Blurred | 57 | 22.90 | <.001** | 3.01 |

**Table S5**

*Descriptive statistics (mean, standard deviation, confidence interval) for posterior the iABR, the centro-parietal iBBR and the midfrontal and posterior iTBR [*µV*] for both modalities and all stimulus types*

|  | *M* | *SD* | Confidence interval | |
| --- | --- | --- | --- | --- |
|  |  |  | Lower limit | Upper limit |
| **iABR** |  |  |  | |
| *PC* |  |  |  | |
| Face | -10.33 | 13.4 | -13.9 | -6.7 |
| Car | -8.13 | 10.9 | -11.1 | -5.2 |
| Face Blurred | -9.65 | 12.9 | -13.1 | -6.2 |
| Car Blurred | -7.60 | 11.7 | -10.8 | -4.4 |
| *VR* |  |  |  |  |
| Face | -11.78 | 12.9 | -15.3 | -8.3 |
| Car | -9.43 | 10.0 | -12.1 | -6.7 |
| Face Blurred | -11.89 | 12.9 | -15.4 | -8.4 |
| Car Blurred | -11.38 | 10.8 | -14.3 | -8.5 |
| **iBBR** |  |  |  | |
| *PC* |  |  |  | |
| Face | 6.48 | 5.3 | 5.1 | 7.9 |
| Car | 5.37 | 4.2 | 4.2 | 6.5 |
| Face Blurred | 5.22 | 4.8 | 3.9 | 6.5 |
| Car Blurred | 5.50 | 4.0 | 4.4 | 6.6 |
| *VR* |  |  |  |  |
| Face | 5.84 | 5.6 | 4.3 | 7.3 |
| Car | 5.23 | 4.6 | 4.0 | 6.5 |
| Face Blurred | 5.86 | 4.9 | 4.5 | 7.2 |
| Car Blurred | 5.02 | 4.4 | 3.8 | 6.2 |
| **Mid-frontal iTBR** |  |  |  | |
| *PC* |  |  |  | |
| Face | 10.60 | 6.0 | 8.7 | 12.5 |
| Car | 8.93 | 6.5 | 7.2 | 10.7 |
| Face Blurred | 9.91 | 6.7 | 8.1 | 11.7 |
| Car Blurred | 9.19 | 6.3 | 7.5 | 10.9 |
| *VR* |  |  |  |  |
| Face | 7.80 | 6.9 | 5.9 | 9.7 |
| Car | 7.83 | 6.9 | 6.0 | 9.7 |
| Face Blurred | 9.72 | 6.8 | 7.9 | 11.6 |
| Car Blurred | 7.90 | 8.4 | 5.6 | 10.2 |
| **Posterior iTBR** |  |  |  | |
| *PC* |  |  |  | |
| Face | 3.80 | 6.5 | 2.1 | 5.6 |
| Car | 2.77 | 5.7 | 1.2 | 4.3 |
| Face Blurred | 3.02 | 5.1 | 1.6 | 4.4 |
| Car Blurred | 3.18 | 5.3 | 1.8 | 4.6 |
| *VR* |  |  |  |  |
| Face | 8.30 | 10.2 | 5.5 | 11.1 |
| Car | 1.76 | 7.8 | -.4 | 3.9 |
| Face Blurred | 3.00 | 7.8 | 1.0 | 5.1 |
| Car Blurred | 1.63 | 7.8 | -.5 | 3.7 |
